# Supplementary material for: Axl and MerTK regulate synovial inflammation and are modulated by IL-6 inhibition in rheumatoid arthritis
Source: Nat Commun. 2024 Mar 16;15:2398. doi: 10.1038/s41467-024-46564-6 (PMC10944458; doi:10.1038/s41467-024-46564-6)
Supplement: Supplementary file 1 — Supplementary Information [file 41467_2024_46564_MOESM1_ESM.pdf]

Axl and MerTK regulate synovial inflammation and are modulated by IL-6 inhibition in rheumatoid arthritis

Alessandra Nerviani[‡1], Marie-Astrid Boutet[‡1,2], Giulia Maria Ghirardi[‡1], Katriona Goldmann[1], Elisabetta Sciacca[1], Felice Rivellese[1], Elena Pontarini[1], Edoardo Prediletto[1], Federico Abatecola[1], Mattia Caliste[1], Sara Pagani[1], Daniele Mauro[1], Mattia Bellan[1,3], Cankut Cubuk[1], Rachel Lau[1], Sarah E. Church[4], Briana M. Hudson[4], Frances Humby[1], Michele Bombardieri[1], Myles J. Lewis[1], Costantino Pitzalis*[1,5]

‡ AN, MAB, and GMG equally contributed

* Corresponding author

Professor Costantino Pitzalis

Centre for Experimental Medicine and Rheumatology, William Harvey Research Institute, Barts and The London School of Medicine and Dentistry, Queen Mary University of London Barts Health NHS Trust, Charterhouse Square, EC1M 6BQ, London, UK

AFFILIATIONS

[1] Centre for Experimental Medicine and Rheumatology, William Harvey Research Institute, Barts and The London School of Medicine and Dentistry, Queen Mary University of London & NIHR BRC Barts Health NHS Trust, London, UK

[2] Nantes Université, Oniris, INSERM, Regenerative Medicine and Skeleton, RMeS, UMR 1229, F-44000 Nantes, France.

[3] Department of Rheumatology, University of Eastern Piedmont and Maggiore della Carita Hospital, Novara, Italy

[4] NanoString Technologies Inc., Seattle, WA, USA

[5] Department of Biomedical Sciences, Humanitas University & IRCCS Humanitas Research Hospital, Milan, Italy

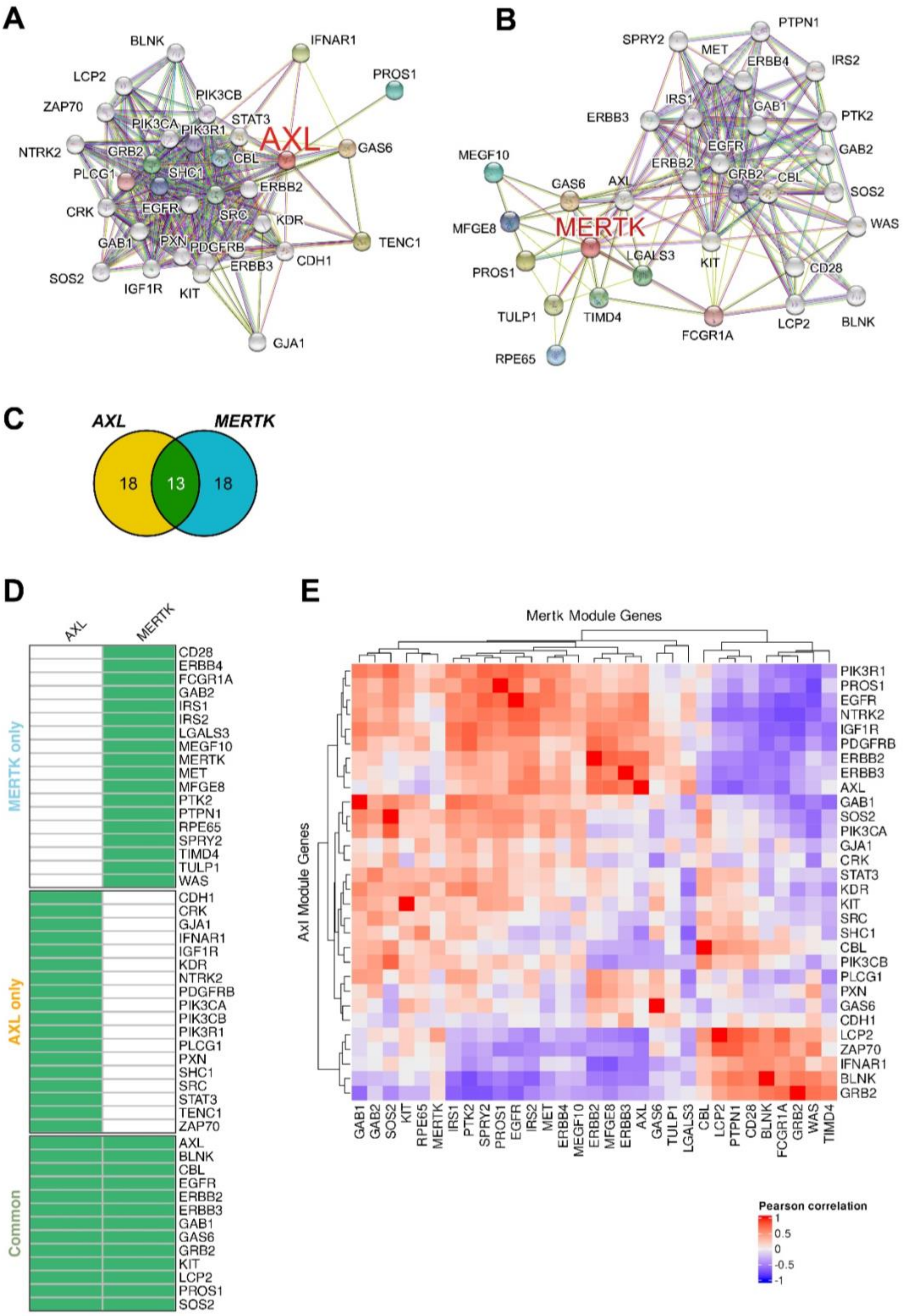

<u>Supplementary Figure 1</u> ***AXL*** **and** ***MERTK*** **STRING networks partially overlap.**

(A, B) STRING functional *AXL* **(A)** and *MERTK* **(C)** protein network (https://string-db.org/). **(C)** Venn's diagram showing overlapping *versus AXL*- and *MERTK*-specific partner genes. **(D)** Detailed list of genes in the *AXL* and *MERTK* networks. **(E)** Heatmap showing the Pearson correlation coefficient between all genes in the *AXL* and *MERTK* modules.

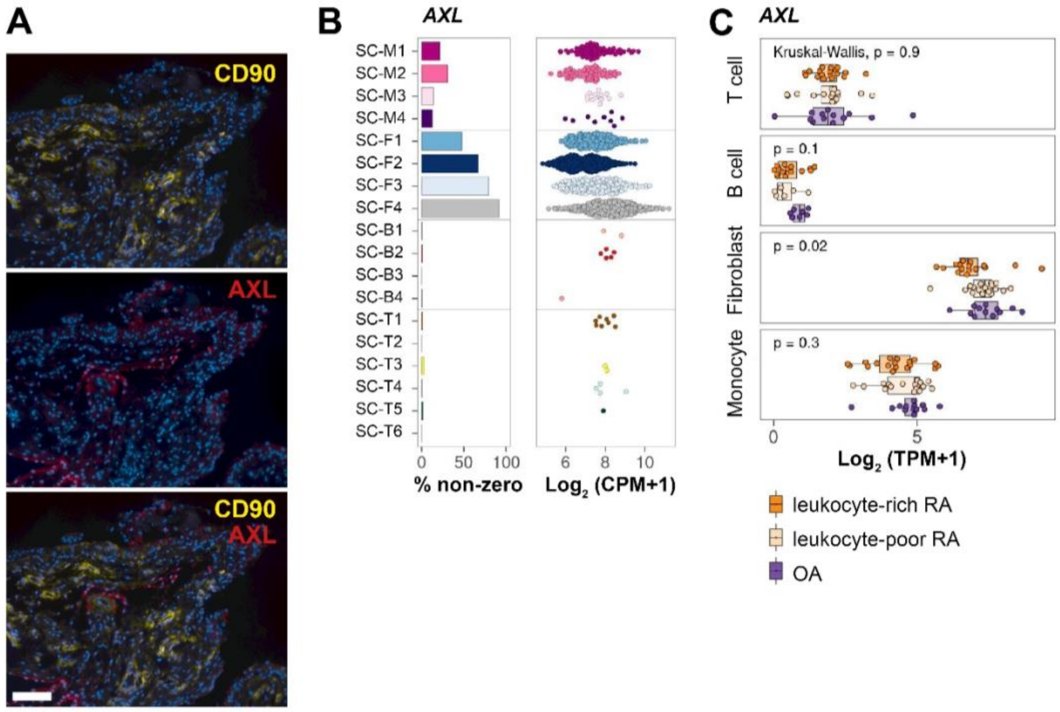

<u>Supplementary Figure 2</u> Axl expression in fibroblasts and scRNA-seq/bulk RNA-seq expression in public databases.

(A) Double immunostaining of CD90 (yellow) and Axl (red) in the synovium of RA patients showing no co-localization between both proteins. Nuclei were counterstained with DAPI (blue). Scale bar = 50µm. Images representative of n=9. **(B, C)** Single-cell **(B)** and bulk RNA-seq **(C)** data downloaded from https://immunogenomics.io/ampra/ [9] showing the expression of *AXL* in B cells (SC-B1 to B4), T cells (SC-T1 to T4), fibroblasts (SC-F1 to F4), and monocytes (SC-M1 to M4) and in leukocyte-rich, leukocyte-poor and osteoarthritis synovial tissue.

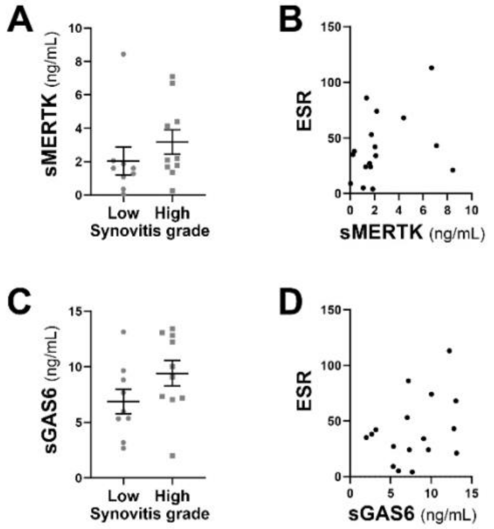

Supplementary Figure 3 **sMerTK and sGas6 expression in the synovial fluid from RA patients according to synovitis score and ESR.**

(A, C) Levels of soluble MerTK (sMerTK) and soluble Gas6 (sGas6) in ng/mL assessed by ELISA in the synovial fluid of RA patients (n=18) divided according to synovitis score (low [0-4], high [5-9]). Data are represented as mean +/- SEM. p-values (not significant) were calculated using the two-tailed Mann Whitney **(B, D)** Correlation between sMerTK **(B)** or sGas6 **(D)** synovial fluid levels and the erythrocyte sedimentation rate (ESR) of RA patients (n=18). p-values (n. s.) calculated according to the two-tailed Spearman correlation test.

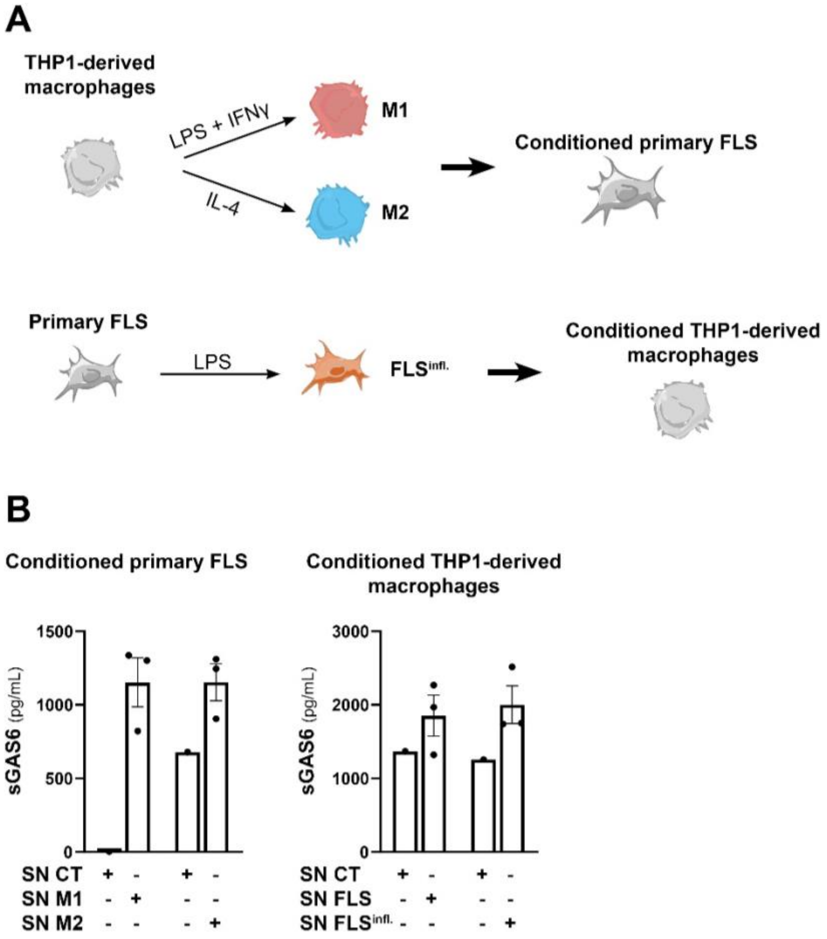

<u>Supplementary Figure 4</u> ***In vitro*** **conditioning experiments.**

(A) Schematic representation of the primary synovial fibroblasts (FLS) and macrophages conditioning experiment. Illustrations were created using Servier medical art [smart.servier.com] under Creative Commons Attribution 3.0 Unported License. **(B)** Expression of Gas6 (pg/mL) in the supernatant of primary fibroblasts-like synoviocytes (FLS) conditioned with supernatant from M1-polarized THP1 (SN M1) or M2-polarized THP1 (SN M2), or in the respective medium used to conditioned the cells (SN CT) (left panel), or in the supernatant of THP1-derived macrophages conditioned with supernatant from unstimulated FLS (SN FLS) or LPS-stimulated FLS (SN FLS Infl.), or in the respective medium used to conditioned the cells (SN CT) (right panel). Data are represented as mean +/- SEM. p-values (n. s.) were calculated using the unpaired two-tailed t-test. Experiments were performed on n=3 distinct patient-derived FLS.

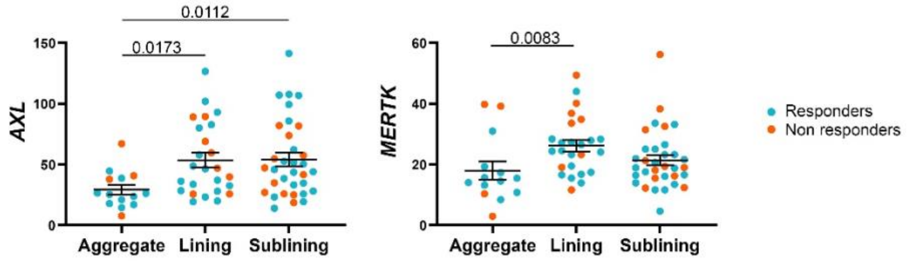

<u>Supplementary Figure 5</u> ***AXL*** **and** ***MERTK*** **RNA expression varies in distinct synovial regions.**

AXL and *MERTK* RNA are differentially expressed in distinct areas of the synovium as quantified by Digital Spatial Profiling (DSP). Both *AXL* and *MERTK* are significantly upregulated in lining and sublining compared to lymphocytic aggregates. Data are represented as mean +/- SEM. p-values were calculated using the Kruskal–Wallis test with Dunn's post-test. N=14 aggregates, including 4 non-responders, 10 responders; n= 25 lining, including 8 non-responders and 17 responders; n=33 sublining, including 12 non-responders and 21 responders.

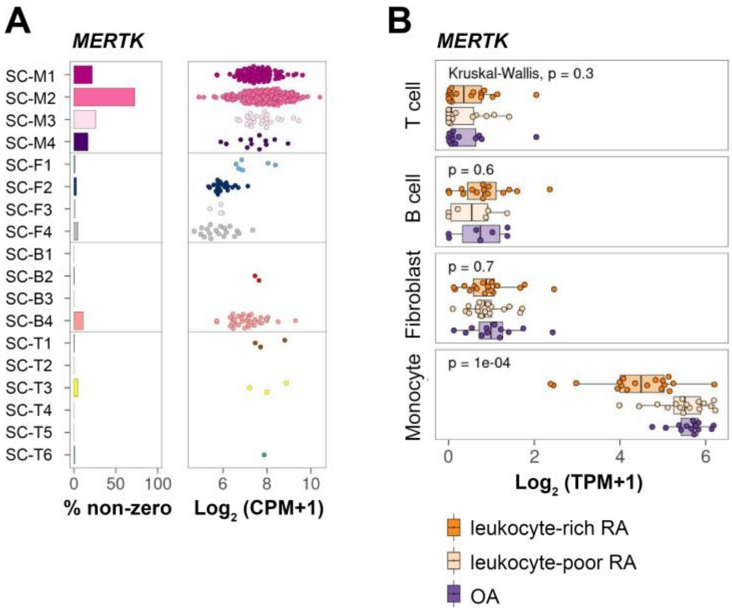

<u>Supplementary Figure 6</u> *MERTK* expression in scRNA-seq and bulk RNA-seq public databases.

Single-cell and bulk RNA-seq data downloaded from https://immunogenomics.io/ampra/ [9] showing the expression of *MERTK* in B cells (SC-B1 to B4), T cells (SC-T1 to T4), fibroblasts (SC-F1 to F4), and monocytes (SC-M1 to M4), and in leukocyte-rich, leukocyte-poor and osteoarthritis synovial tissue.

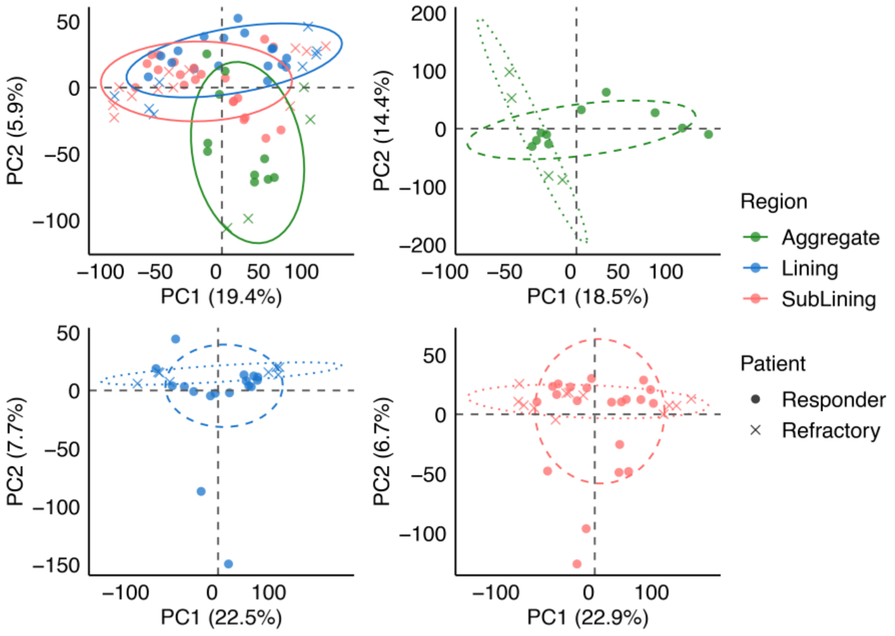

Supplementary Figure 7 Clustering of the regions of interest in synovial biopsies from rheumatoid arthritis patients.

Principal component analysis (PCA) of the digital spatial profiling (DSP) of 72 regions of interest (aggregate, lining and sublining) in synovial biopsies from rheumatoid arthritis patients (4 refractory and 8 responders to rituximab or tocilizumab).

RNA-Seq Cohort (n = 87)

**Demographics**		
Female, % (n)		73.6% (64)
Age, years (mean ± SD)		53.6 ± 16.7
Disease duration, months (mean ± SD)		5.9 ± 3.2
**Biochemical and clinical parameters**		
ESR, mm/hr (mean ± SD)		.44.6 ± 29.4
CRP, mg/l (mean ± SD)		22.8 ± 32.9
RF+, % (n)		.71.3% (62)
Anti-CCP+, % (n)		73.6 % (64)
RF and/or anti-CCP+, % (n)		78.2% (68)
TJ/28 joints (mean ± SD)		12.1 ± 7.6
SJ/28 joints (mean ± SD)		.7.9 ± 5.7
VAS GH patient (mean ± SD)		65 ± 24
DAS28 (mean ± SD)		5.9 ± 1.3
**Synovial biopsy site**		
.Biopsied joint	MCP/PIP/MTP, % (n)	.13.8% (12)
	Wrist, % (n)	63.2% (55)
	Knee, % (n)	.23% (20)
**Histology**		
Synovitis score [1]		4.7 ± 2.3

<u>Supplementary Table 1</u> Baseline clinical characteristics of the patients included in the Pathobiology of Early Arthritis Cohort (PEAC) for RNA-seq analysis (n=87).

n, number; SD, standard deviation; ESR, erythrocyte sedimentation rate; CRP, C-Reactive Protein; RF, rheumatoid factor; CCP, cyclic citrullinated peptide; TJC/28, tender joints count (0-28); SJC/28, swollen joints count/28; VAS GH, Visual Analogue Scale General Health (0-100); DAS28, Disease Activity Score 28; MCP, metacarpophalangeal joints; PIP, proximal interphalangeal joints; MTP, metatarsophalangeal joints.

[1] 10 synovial tissue samples were classified as ungraded. Synovitis score was available for 77 patients.

Synovial Fluid Cohort (n = 18)

**Demographics**	
Female, % (n)	72.2 % (13)
Age, years (mean ± SD)	52.3 ± 15.6
Disease duration, months (mean ± SD)	4.9 ± 2.1
**Biochemical and clinical parameters**	
ESR, mm/hr (mean ± SD)	.41.2 ± 29.7
CRP, mg/l (mean ± SD)	23.6 ± 21.5
RF+, % (n) [1]	.86.7 % (13)
Anti-CCP+, % (n) [1]	80 % (12)
RF and/or anti-CCP+, % (n) [1]	86.7 % (13)
TJ/28 joints (mean ± SD) [2]	9.3 ± 6.3
SJ/28 joints (mean ± SD) [2]	.6.4 ± 5.3
VAS GH patient (mean ± SD) [2]	76.3 ± 17.5
DAS28 (mean ± SD) [2]	5.6 ± 1
**Histology**	
Synovitis score	5.3 ± 2.5

Supplementary Table 2 Baseline clinical characteristics of the patients included in the Pathobiology of Early Arthritis Cohort (PEAC) for synovial fluid ELISA analysis (n=18).

n, number; SD, standard deviation; ESR, erythrocyte sedimentation rate; CRP, C-Reactive Protein; RF, rheumatoid factor; CCP, cyclic citrullinated peptide; TJC/28, tender joints count (0-28); SJC/28, swollen joints count/28; VAS GH, Visual Analogue Scale General Health (0-100); DAS28, Disease Activity Score 28; MCP, metacarpophalangeal joints; PIP, proximal interphalangeal joints; MTP, metatarsophalangeal joints.

[1] Anti-CCP and RF status available for 15/18 patients.

[2] Number of tender and swollen joints, VAS GH patient and DAS28 available for 17/18 patients
