# Supplementary material for: Axl and MerTK regulate synovial inflammation and are modulated by IL-6 inhibition in rheumatoid arthritis
Source: Nat Commun. 2024 Mar 16;15:2398. doi: 10.1038/s41467-024-46564-6 (PMC10944458; doi:10.1038/s41467-024-46564-6)
Supplement: Supplementary file 3 — Reporting Summary [file 41467_2024_46564_MOESM3_ESM.pdf]

nature portfolio

Corresponding author(s): Costantino Pitzalis

Last updated by author(s): 14/02/2024



# Reporting Summary

Nature Portfolio wishes to improve the reproducibility of the work that we publish. This form provides structure for consistency and transparency in reporting. For further information on Nature Portfolio policies, see our Editorial Policies and the Editorial Policy Checklist.

## Statistics

For all statistical analyses, confirm that the following items are present in the figure legend, table legend, main text, or Methods section.

| n/a | Confirmed | |
|---|---|---|
| [ ] | [x] | The exact sample size (*n*) for each experimental group/condition, given as a discrete number and unit of measurement |
| [ ] | [x] | A statement on whether measurements were taken from distinct samples or whether the same sample was measured repeatedly |
| [ ] | [x] | The statistical test(s) used AND whether they are one- or two-sided<br>*Only common tests should be described solely by name; describe more complex techniques in the Methods section.* |
| [ ] | [x] | A description of all covariates tested |
| [ ] | [x] | A description of any assumptions or corrections, such as tests of normality and adjustment for multiple comparisons |
| [ ] | [x] | A full description of the statistical parameters including central tendency (e.g. means) or other basic estimates (e.g. regression coefficient) AND variation (e.g. standard deviation) or associated estimates of uncertainty (e.g. confidence intervals) |
| [ ] | [x] | For null hypothesis testing, the test statistic (e.g. *F*, *t*, *r*) with confidence intervals, effect sizes, degrees of freedom and *P* value noted<br>*Give P values as exact values whenever suitable.* |
| [x] | [ ] | For Bayesian analysis, information on the choice of priors and Markov chain Monte Carlo settings |
| [x] | [ ] | For hierarchical and complex designs, identification of the appropriate level for tests and full reporting of outcomes |
| [ ] | [x] | Estimates of effect sizes (e.g. Cohen's *d*, Pearson's *r*), indicating how they were calculated |

*Our web collection on statistics for biologists contains articles on many of the points above.*

## Software and code

Policy information about availability of computer code

Data collection

When available, 1µg total RNA was used for library preparation using either TruSeq RNA Sample Preparation Kit v2 for Illumina (PEAC) or NEBNext Ultra RNA Library Prep kit for Illumina (R4RA cohort). The libraries were multiplexed and then sequenced on an Illumina HiSeq instrument as per manufacturer's instructions.
NanoString GeoMx WTA sequencing reads were compiled into FASTQ files corresponding to each ROI. FASTQ files were converted to digital count conversion files using the NanoString NanoString GeoMx NGS DnD Pipeline. Q3 normalisation was performed as described in Rivellese, F. et al. Rituximab versus tocilizumab in rheumatoid arthritis: synovial biopsy-based biomarker analysis of the phase 4 R4RA randomized trial. Nat Med 28, 1256-1268 (2022).

Data analysis

RNAseq analyses: Raw data quality control, normalisation, alignment, and analysis of normalised log expression read counts were performed as previously described in Lewis, M. J. et al. Molecular Portraits of Early Rheumatoid Arthritis Identify Clinical and Treatment Response Phenotypes. Cell Rep 28, 2455-2470.e5 (2019) and Rivellese, F. et al. Rituximab versus tocilizumab in rheumatoid arthritis: synovial biopsy based biomarker analysis of the phase 4 R4RA randomized trial. Nat Med 28, 1256-1268 (2022).
NanoString GeoMx analyses: A mixed effects model was fitted via Ime4 (v1.1-27.1) using the ROI location as fixed effects and patients IDs as random effects. One-way ANOVA and pairwise tests between ROIs were derived via the car package (v3.0-12) and used to show differential and overlapping genes across aggregate, lining and sublining regions in a three-way radial plot made via volcano3D (v1.3.1). Normalised expression of selected individual genes was represented in boxplots using ggplot2 (v3.3.6). Principal component analysis (PCA) on the normalised data was undertaken using prcomp from the stats package (v4.2.0) and plotted using ggplot2(v3.3.6).
Gene set enrichment analysis (GSEA) was performed using the R interface (v3.0.0) (https://CRAN.R-project.org/package=enrichR).
Module genes were selected by creating STRING networks (Szklarczyk, D. et al. The STRING database in 2021: customizable protein—protein networks, and functional characterization of user-uploaded gene/measurement sets. Nucleic Acids Res 49, D6O5-D612 (2021)) centred on

the target gene and extending until more than 31 genes were included in the network. Module scores for RNAseq data were derived by singular value decomposition (SVD) for each gene module matrix using a methodology described in detail in Langfelder, P. & Horvath, S. Eigengene networks for studying the relationships between co-expression modules. BMC Syst Biol 1, 54 (2007). STRING homo sapiens database was accessed via https://string-db.org/ on 16th August 2021

For manuscripts utilizing custom algorithms or software that are central to the research but not yet described in published literature, software must be made available to editors and reviewers. We strongly encourage code deposition in a community repository (e.g. GitHub). See the Nature Portfolio guidelines for submitting code & software for further information.

## Data

Policy information about availability of data

All manuscripts must include a data availability statement. This statement should provide the following information, where applicable:

- Accession codes, unique identifiers, or web links for publicly available datasets
- A description of any restrictions on data availability
- For clinical datasets or third party data, please ensure that the statement adheres to our policy

The PEAC data used in this study have been deposited in the ArrayExpress database under accession code E-MTAB-6141 [https://www.ebi.ac.uk/biostudies/arrayexpress/studies/E-MTAB-6141].
The R4RA data used in this study have been deposited in the ArrayExpress database under accession code MTAB-11611 [https://www.ebi.ac.uk/biostudies/arrayexpress/studies/ MTAB-11611].
The processed PEAC data are available on https://peac.hpc.qmul.ac.uk/ as interactive web interface that allow direct data exploration as published by Lewis, M. J. et al. Molecular Portraits of Early Rheumatoid Arthritis Identify Clinical and Treatment Response Phenotypes. Cell Rep 28, 2455-2470.e5 (2019).
The processed R4RA data are available on https://r4ra.hpc.qmul.ac.uk/ as interactive web interface that allow direct data exploration as published by and Rivellese, F. et al. Rituximab versus tocilizumab in rheumatoid arthritis: synovial biopsy based biomarker analysis of the phase 4 R4RA randomized trial. Nat Med 28, 1256-1268 (2022).
STRING homo sapiens database was accessed via https://string-db.org/ on 16th August 2021

## Research involving human participants, their data, or biological material

Policy information about studies with human participants or human data. See also policy information about sex, gender (identity/presentation), and sexual orientation and race, ethnicity and racism.

| | |
|---|---|
| Reporting on sex and gender | We reported sex of human participants in Methods. Sex was collected from medical records. We could not analyze disaggregated data. Findings are applicable to any sex. Higher number of female subjects reflect the epidemiology of the disease in study. |
| Reporting on race, ethnicity, or other socially relevant groupings | Specific data on race, ethnicity or socially groupings were not available. |
| Population characteristics | Population characteristics have been reported in Supplementary Table 1 and Supplementary Table 2 |
| Recruitment | Participant were recruited after obtaining written informed consent and leaving at least 24h period after providing them with the PIS as per Good Medical Practice (GCP) guidelines.<br>Detailed recruitment procedures have been described in and Humby F, Lewis M, Ramamoorthi N, et al. Synovial cellular and molecular signatures stratify clinical response to csDMARD therapy and predict radiographic progression in early rheumatoid arthritis patients. Ann Rheum Dis. 2019;78(6):761-772. doi:10.1136/annrheumdis-2018-214539 and Humby F, Durez P, Buch MH, et al. Rituximab versus tocilizumab in anti-TNF inadequate responder patients with rheumatoid arthritis (R4RA): 16-week outcomes of a stratified, biopsy-driven, multicentre, open-label, phase 4 randomised controlled trial. Lancet. 2021;397(10271):305-317. doi:10.1016/S0140-6736(20)32341-2 |
| Ethics oversight | National Research Ethics Service Committee London Dulwich (REC 05/C10703/198) and UK Medical Research and Ethics Committee (MREC) reference: 12/WA/0307 |

Note that full information on the approval of the study protocol must also be provided in the manuscript.

# Field-specific reporting

Please select the one below that is the best fit for your research. If you are not sure, read the appropriate sections before making your selection.

[x] Life sciences  [ ] Behavioural & social sciences  [ ] Ecological, evolutionary & environmental sciences

For a reference copy of the document with all sections, see nature.com/documents/nr-reporting-summary-flat.pdf

# Life sciences study design

All studies must disclose on these points even when the disclosure is negative.

| | |
|---|---|
| Sample size | We report ad hoc analyses of two previously published clinical studies:<br>1) PEAC, described in Humby F, Lewis M, Ramamoorthi N, et al. Synovial cellular and molecular signatures stratify clinical response to |

csDMARD therapy and predict radiographic progression in early rheumatoid arthritis patients. Ann Rheum Dis. 2019;78(6):761-772. doi:10.1136/annrheumdis-2018-214539 and Lewis, M. J. et al. Molecular Portraits of Early Rheumatoid Arthritis Identify Clinical and Treatment Response Phenotypes. Cell Rep 28, 2455-2470.e5 (2019). No sample size calculation was performed a priori as this is an observational study. In this manuscript, we analysed data from the first sequential RA patients, who had been RNA-sequenced (n=87, of whom 73.6% female, for RNA-seq analysis; n=18, of whom 72.2 % female, for synovial fluid analysis).
2) R4RA, described in Humby F, Durez P, Buch MH, et al. Rituximab versus tocilizumab in anti-TNF inadequate responder patients with rheumatoid arthritis (R4RA): 16-week outcomes of a stratified, biopsy-driven, multicentre, open-label, phase 4 randomised controlled trial. Lancet. 2021;397(10271):305-317. doi:10.1016/50140-6736(20)32341-2 and Rivellese, F. et al. Rituximab versus tocilizumab in rheumatoid arthritis: synovial biopsy-based biomarker analysis of the phase 4 R4RA randomized trial. Nat Med 28, 1256-1268 (2022).
According to the power calculation of the R4RA trial, a sample size of 82 B-cell-poor patients was assessed to provide 90% power to detect a 35% difference (assuming 55% response rate to Tocilizumab and 20% in Rituximab determined in previously conducted pilot study) in the proportion of patients who were deemed as responders by the primary endpoint (improvement in CDAI score of at least 50% at week 16). After estimating that 10% of biopsy samples would be ungradable and assuming a 5% dropout rate, a total of 160 patients would be required to recruit 82 patients who were B-cell poor. n= 161 synovial samples were available at baseline and n=65 at 16 weeks. For molecular analyses (RNA-seq), following quality control, n= 133 R4RA samples (whole cohort) were available at baseline and n=44 at 16 weeks.
For in vitro experiments and IF staining, at least n=3 biological replicates have been used to ensure statistical significance and consistency in the results in different patients/pathotypes.

Data exclusions

1) PEAC (as reported in Lewis, M. J. et al. Molecular Portraits of Early Rheumatoid Arthritis Identify Clinical and Treatment Response Phenotypes. Cell Rep 28, 2455-2470.e5 (2019)).
90 sequential synovial samples acquired through a minimally invasive US-guided synovial biopsy were RNA-seq; three synovium RNA sample were outliers, transcript abundances for the remaining synovial samples (n = 87) were anaysed and presented here.
2) R4RA (as reported in Rivellese, F. et al. Rituximab versus tocilizumab in rheumatoid arthritis: synovial biopsy-based biomarker analysis of the phase 4 R4RA randomized trial. Nat Med 28, 1256-1268 (2022)).
All the analyses presented were done in the intention-to-treat population. 164 patients were randomised but 3 patients did not receive the study drug, so were excluded from the intention-to-treat population. All baseline (n=161) and 16 weeks (n=65) synovial samples were sent for RNA-seq. Following RNA-Seq quality control 36 samples were excluded due to poor mapping or RNA quality. and one outlier was identified and removed from further analysis. 133 patients had RNA-Seq data available for subsequent analysis at baseline and 44 patients for the follow-up time point.

Re: in vitro experiments, no data have been excluded.

Replication

For all patients (PEAC and R4RA), a minimum of 6 synovial samples were assessed by histology to determine the pathotype, and a minimum of 6 samples were pooled for RNA extraction and RNA sequencing.
In vitro experiments were performed on a minimum of n=3 biological replicates (in technical duplicates). All ELISAs were performed as duplicates.

Randomization

PEAC did not require randomization since patients were treated as per standard of care.
R4RA: As described in Humby F, Durez P, Buch MH, et al. Rituximab versus tocilizumab in anti-TNF inadequate responder patients with rheumatoid arthritis (R4RA): 16-week outcomes of a stratified, biopsy-driven, multicentre, open-label, phase 4 randomised controlled trial. Lancet. 2021;397(10271):305-317. doi:10.1016/50140-6736(20)32341-2, at week 0, patients were randomly assigned (1:1) in block sizes of six and four to the rituximab group or the tocilizumab group stratified into four blocks according to histological classification of baseline synovial biopsy (B-cell poor, B-cell rich, germinal centre positive, or unknown) and by site (Queen Mary University London, London, UK vs all other sites) using an interactive web response system. More details on randomization are available in the publication reporting the primary trial results (Humby et al, Lancet 2021).

Blinding

PEAC did not require blinding.
In R4RA, investigators and patients were blinded to the synovial pathotype, however the Ethics Committee advised against double-blinding the trial because it would be impractical and extremely inconvenient for patients. Since tocilizumab is given as monthly infusion, compared with rituximab, given every 6 months, blinding would have required all patients to have monthly infusions.

# Reporting for specific materials, systems and methods

We require information from authors about some types of materials, experimental systems and methods used in many studies. Here, indicate whether each material, system or method listed is relevant to your study. If you are not sure if a list item applies to your research, read the appropriate section before selecting a response.

## Materials & experimental systems

| n/a | Involved in the study | |
|---|---|---|
| ☐ | ☒ | Antibodies |
| ☐ | ☒ | Eukaryotic cell lines |
| ☒ | ☐ | Palaeontology and archaeology |
| ☒ | ☐ | Animals and other organisms |
| ☐ | ☒ | Clinical data |
| ☒ | ☐ | Dual use research of concern |
| ☒ | ☐ | Plants |

## Methods

| n/a | Involved in the study | |
|---|---|---|
| ☒ | ☐ | ChIP-seq |
| ☒ | ☐ | Flow cytometry |
| ☒ | ☐ | MRI-based neuroimaging |

## Antibodies

| | |
|---|---|
| Antibodies used | For immunohistochemistry, the following Ab were used: CD68 (Dako, M0814 , KP1 , Mouse IgG, 1:50), CD3 (Dako, M7254, F7.2.38, Mouse IgG1 kappa, 1:50), CD20 (Dako, M0755, L26, Mouse IgG2a kappa, 1:50), CD138 (Dako, M7228, MI15, Mouse IgG1 kappa, 1:50), Axl (R&D Systems, AF154, Polyclonal, Goat IgG, 1:200), MerTK (Abcam, ab52968, Y323, Rabbit IgG, 1:500), VisUCyteTM anti-Goat, R&D Systems , VC004-025, Polyclonal, Donkey anti-Goat IgG, NA (ready-to-use), Envision+ System-HRP labelled anti-rabbit, Dako, K4003, Polyclonal, Goat anti- rabbit, NA (ready-to-use), EnVision+ System-HRP labelled anti-mouse, Dako , K4001, Polyclonal, Goat anti-mouse, NA (ready-to-use).<br>For multiple Immunofluorescence staining, the following Ab were used: CD68 (Dako, M0814 , KP1 , Mouse IgG, 1:50), Axl (R&D Systems, AF154, Polyclonal, Goat IgG, 1:200), MerTK (Abcam, ab52968, Y323, Rabbit IgG, 1:500), ADAM10 (Abcam, ab124695, EPR5622, Rabbit IgG, 1:100), CD55 (Abcam, ab133684, EPR6689, Rabbit IgG, 1:100), CD90 (Abcam, ab133350, EPR3133, Rabbit IgG, 1:240); AlexaFluor488 (Cat:B40953), AlexaFluor555 (B40955), AlexaFluor647 (B40958) all from Invitrogen, ThermoFisher Scientific, 1:200.<br>For Nanostring GeoMX, the following antibodies were used: CD68-AF532 (clone KP1, Cat. N. NB100-683 Novus), CD20-DL594 (clone IGEL/773, Cat. N. NBP2-44745, Novus), and CD3-AF647 (clone UMAB54, Cat. N. TA807198, Origene). |
| Validation | All antibodies used for IHC and IF are commercially available and have been validated by the producer for use in IHC/IF on FFPE human tissue.<br>- CD68 (Dako/Agilent, M0814): https://www.agilent.com/en/product/immunohistochemistry/antibodies-controls/primary-antibodies/cd68-%28dako-omnis%29-76223<br>- CD68(Novus, clone KP1): https://www.novusbio.com/products/cd68-sr-d1-antibody-kp1_nb100-683af532<br>- CD3 (Dako/Agilent, M7254): https://www.agilent.com/en/product/immunohistochemistry/antibodies-controls/primary-antibodies/cd3-%28concentrate%29-76649<br>- CD3 (Origene, Cat. N. TA807198) https://www.origene.com/catalog/antibodies/primary-antibodies/ta807198/cd3e-mouse-monoclonal-antibody-clone-id-ucht1<br>- CD2O (Dako/Agilent, M0755): https://www.agilent.com/en/product/immunohistochemistry/antibodies-controls/primary-antibodies/cd20cy-%28concentrate%29-76520<br>- CD20 (Novus, clone IGEL/773) https://www.novusbio.com/products/cd20-antibody-igel-773_nbp2-44745<br>- CD138: https://www.agilent.com/en/product/immunohistochemistry/antibodies-controls/primary-antibodies/cd138-%28concentrate%29-76642.<br>- Axl: https://www.rndsystems.com/products/human-axl-antibody_af154<br>- MerTK: https://www.abcam.com/products/primary-antibodies/mertk-antibody-y323-ab52968.html<br>- ADAM10: https://www.abcam.com/products/primary-antibodies/adam10-antibody-epr5622-ab124695.html<br>- CD55: https://www.abcam.com/products/primary-antibodies/cd55-antibody-epr6689-ab133684.html<br>- CD9O: https://www.abcam.com/products/primary-antibodies/cd90--thy1-antibody-epr3133-ab133350.html<br><br>All DAKO/Agilent antibodies have been validated by the producer for use in human pathology:<br>The synovial CD20 staining has been further validated in Rivellese F, et al. Arthritis Rheumatol. 2020. (https://doi.org/10.1002/art.41184).<br><br>In addition:<br>- MerTK (clone Y323) has been used in synovia by Alivernini S et al, Distinct synovial tissue macrophage subsets regulate inflammation and remission in rheumatoid arthritis. Nat Med. 2020 Aug;26(8):1295-1306. doi: 10.1038/s41591-020-0939-8)<br>- CD90 has been used in synovia by Stephenson, W et al., Single-cell RNA-seq of rheumatoid arthritis synovial tissue using low-cost microfluidic instrumentation. Nat Commun 9, 791 (2018). https://doi.org/10.1038/s41467-017-02659-x and Rivellese, F et al. Rituximab versus tocilizumab in rheumatoid arthritis: synovial biopsy-based biomarker analysis of the phase 4 R4RA randomized trial. Nat Med 28, 1256-1268 (2022).<br><br>All antibodies described here have been further optimized for use in synovia by testing several dilutions and using isotype controls. |

## Eukaryotic cell lines

Policy information about cell lines and Sex and Gender in Research

| | |
|---|---|
| Cell line source(s) | Name: THP-1 is a monocyte isolated from peripheral blood from an acute monocytic leukemia patient (male).<br>Primary fibroblast-like-synoviocytes (FLS) (n=3, with n=2 male and n=1 female) were used between P3 and P10 (not immortalized) |
| Authentication | Authentication: As found in the supplier's page https://www.atcc.org/products/tib-202<br>As per supplier, validation performed by STR Profiling |
| Mycoplasma contamination | Mycoplasma: THP1 tested negative for Mycoplasma contamination. |
| Commonly misidentified lines<br>(See ICLAC register) | N/A |

## Clinical data

Policy information about clinical studies

All manuscripts should comply with the ICMJE guidelines for publication of clinical research and a completed CONSORT checklist must be included with all submissions.

| | |
|---|---|
| Clinical trial registration | "Rituximab versus tocilizumab in anti-TNF inadequate responder (ir) patients with rheumatoid arthritis" (R4RA)<br>UK Medical Research and Ethics Committee (MREC) reference: 12/WA/0307 |
| Study protocol | Available at www.r4ra-nihr.whri.qmul.ac.uk/docs/r4ra_protocol_version_9_30.10.2017_clean.pdf |
| Data collection | Between Feb 28, 2013, and Jan 17, 2019<br>Detailed clinical trial procedures published here Humby F, Durez P, Buch MH, et al. Rituximab versus tocilizumab in anti-TNF inadequate responder patients with rheumatoid arthritis (R4RA): 16-week outcomes of a stratified, biopsy-driven, multicentre, open-label, phase<br>4 randomised controlled trial. Lancet. 2021;397(10271):305-317. doi:10.1016/S0140-6736(20)32341-2 |
| Outcomes | Please see the main publication for primary and secondary outcomes (Humby et al, Lancet 2021, DOI:https://doi.org/10.1016/S0140-6736(20)32341-2) |

## Plants

| | |
|---|---|
| Seed stocks | *Report on the source of all seed stocks or other plant material used. If applicable, state the seed stock centre and catalogue number. If plant specimens were collected from the field, describe the collection location, date and sampling procedures.* |
| Novel plant genotypes | *Describe the methods by which all novel plant genotypes were produced. This includes those generated by transgenic approaches, gene editing, chemical/radiation-based mutagenesis and hybridization. For transgenic lines, describe the transformation method, the number of independent lines analyzed and the generation upon which experiments were performed. For gene-edited lines, describe the editor used, the endogenous sequence targeted for editing, the targeting guide RNA sequence (if applicable) and how the editor was applied.* |
| Authentication | *Describe any authentication procedures for each seed stock used or novel genotype generated. Describe any experiments used to assess the effect of a mutation and, where applicable, how potential secondary effects (e.g. second site T-DNA insertions, mosiacism, off-target gene editing) were examined.* |
